# School health promotion and fruit and vegetable consumption in secondary schools: a repeated cross-sectional multilevel study

**DOI:** 10.1186/s12889-024-18546-2

**Published:** 2024-04-22

**Authors:** Lisanne Vonk, Iris Eekhout, Tim Huijts, Mark Levels, Maria W.J. Jansen

**Affiliations:** 1 Academic Collaborative Center for Public Health Limburg, Public Health Service South Limburg, 6400 AA Heerlen, P.O. Box 33, the Netherlands; 2 Department of Health Services Research, Care and Public Health Research Institute (CAPHRI), Maastricht University, 6200 MD Maastricht, P.O. Box 616, the Netherlands; 3 Expertise Center Child Health, Netherlands Organisation for Applied Scientific Research (TNO), 2301 DA Leiden, P.O. Box 3005, the Netherlands; 4 Research Centre for Education and the Labour Market (ROA), School of Business and Economics, Maastricht University, Postbus 616, 6200 MD Maastricht, the Netherlands

**Keywords:** School health promotion, Multilevel analysis, Secondary school, Fruit, Vegetable

## Abstract

**Background:**

Worldwide, recommendations for fruit and vegetable consumption are not met, which can cause chronic diseases. Especially adolescence is an important phase for the development of health behaviours. Therefore, in the Netherlands, the Healthy School program was established to aid schools in promoting healthy lifestyles among their students. We examined to what extent the variation between secondary schools regarding students’ fruit and vegetable consumption could be explained by differences between schools regarding Healthy School certification, general school characteristics, and the school population. Additionally, we examined whether Healthy School certification was related to the outcomes, and whether the association differed for subgroups.

**Methods:**

We performed a repeated cross-sectional multilevel study. We used data from multiple school years from the national Youth Health Monitor on secondary schools (grades 2 and 4, age ranged from approximately 12 to 18 years) of seven Public Health Services, and added data with regard to Healthy School certification, general school characteristics and school population characteristics. We included two outcomes: the number of days a student consumed fruit and vegetables per week. In total, we analysed data on 168,127 students from 256 secondary schools in the Netherlands.

**Results:**

Results indicated that 2.87% of the variation in fruit consumption and 5.57% of the variation in vegetable consumption could be attributed to differences at the school-level. Characteristics related to high parental educational attainment, household income, and educational track of the students explained most of the variance between schools. Additionally, we found a small favourable association between Healthy School certification and the number of days secondary school students consumed fruit and vegetables.

**Conclusions:**

School population characteristics explained more variation between schools than Healthy School certification and general school characteristics, especially indicators of parental socioeconomic status. Nevertheless, Healthy School certification seemed to be slightly related to fruit and vegetable consumption, and might contribute to healthier dietary intake. We found small differences for some subgroups, but future research should focus on the impact in different school contexts, since we were restricted in the characteristics that could be included in this study.

**Supplementary Information:**

The online version contains supplementary material available at 10.1186/s12889-024-18546-2.

## Background

Globally, the inadequate consumption of fruit and vegetables continues to pose a public health challenge. In many countries worldwide, recommendations for fruit and vegetable consumption are not met [1, 2], indicating concerning patterns in dietary habits. Fruit and vegetables are rich sources of vitamins, minerals, and fibres, which are important in a healthy diet [3]. A deficit of these nutrients has been related to chronic diseases, such as cardiovascular diseases and various types of cancer [4]. In 2017, insufficient fruit and vegetable consumption caused around 3.9 million deaths worldwide [5]. Especially adolescence is an important phase for the development of health behaviours [6]. Therefore, it is concerning that in the Netherlands, the majority of adolescents over 12 years of age do not meet the national guidelines for fruit and vegetable intake [7, 8]. Diet quality even tends to decrease, as evidence shows that students in secondary schools consume less fruit and vegetables [8, 9].

Worldwide, schools implement programs and interventions based on the Health Promoting Schools (HPS) framework of the World Health Organization (WHO) to promote healthy habits, such as healthier dietary intake, of their students [10]. The HPS framework focuses on six components: policy, physical and social environment, skills and education, the community including parents, and access to health services. However, dietary habits of children are strongly determined by their parents [11]. As children become older, the influence of peers becomes more important, as previous literature results have shown that dietary intake of adolescents is strongly related to descriptive social norms, i.e. beliefs about what others do or how they behave [12, 13]. This raises the question whether secondary schools, for example by implementing health promotion programs, can influence the dietary intake of students. A systematic review of Langford et al. [14] showed that programs based on the HPS framework can be effective in stimulating fruit and vegetable consumption among students. Nevertheless, the authors added to this conclusion that more research is needed to evaluate the impact on health-promoting programs targeting dietary intake in secondary schools, since the majority of studies predominantly focused on children under the age of 13. Additionally, even though positive results were shown, results were inconsistent and observed effects were small [14].

We aim to explore whether we can shed more light on the inconsistent and relatively modest nature of these effects if we focus on the school context. Schools are often considered to be complex adaptive systems, meaning that implementing health-promoting programs or interventions can have different effects [15]. For example, the geographic location of the school area could influence the impact of school health promotion (SHP) due to the proximity of food suppliers [16]. Other potential moderators are the household income and educational attainment of the parents, as they are related to their child’s health [17]. Demographic factors could also be a possible moderator. For example, larger schools might have greater resources to implement health-promoting activities [18]. On the other hand, larger schools also have more staff and students, which might complicate the implementation of SHP [19]. Examining the interaction between the school context and SHP is therefore of great importance to understand the impact of contextual factors [20].

In this paper, we analyse the interplay of various school characteristics in the Netherlands. The aim of this study was to evaluate a whole-school approach, i.e. the Dutch Healthy School (HS) program, which largely aligns with the HPS framework. Therefore, we address two research questions in this study: (1) To what extent can the variation between secondary schools in the Netherlands regarding fruit and vegetable consumption of students be explained by differences between schools regarding SHP, operationalised as HS certification, general school characteristics, and the school population? (2) To what extent is HS certification related to fruit and vegetable consumption of students, and is this relation moderated by general school characteristics and school population characteristics? Both research questions were analysed separately for fruit and vegetable consumption. We hypothesised that HS certification, especially the nutrition certificate, is positively related to the number of days a student consumed fruit and vegetables per week, but that the strength of the association varies due to different contextual factors.

## Methods

### Study design and study population

We performed a repeated cross-sectional multilevel study. The Netherlands has twenty-five regional Public Health Services (PHSs, *GGD’en* in Dutch), of which seven provided anonymised data for this study of 185,138 students. These seven PHSs provided data of the schools in their region that participated in the Youth Health Monitor or its precursor the Electronic Monitor and Health Education (E-MOVO) in the school years 2011–2012 or 2013–2014, 2015–2016, and 2019–2020 (prior to the Covid-19 pandemic). The Youth Health Monitor and E-MOVO are standardised surveys regarding dietary intake and other individual (health) characteristics that are administered among secondary school students. The Youth Health Monitor is administered every four years. Participating secondary school students in their second and fourth year can fill out the survey during school hours and were recruited through passive consent. Their age ranged from approximately 12 to 18 years. Most surveys were filled out anonymously, except for the E-MOVO survey in one PHS. The variables that were included by the PHSs in their survey and that were used for this study could differ per school year [see Additional file 1]. We also used (aggregated) data from the *Netherlands Cohort Study on Education* (NCO), initiated by the Netherlands Initiative for Education Research (NRO). The NCO has data of all secondary schools, except for approximately 50 privately funded schools. These schools were therefore not included in our study [21, 22]. Almost all school-level characteristics could differ per school year and encrypted school identifiers were used to merge datasets.

Students without a known school identifier were excluded (5.6%). We also excluded students from special needs schools (0.2%), students outside grades 2 and 4 (0.3%), and students that were in an educational track other than pre-vocational secondary education (Dutch: *vmbo*), senior general secondary education (Dutch: *havo*), or pre-university education (Dutch: *vwo*) (0.5%). The majority of children in the Netherlands enrolls into secondary school at the age of 12 in either of these educational tracks. Pre-vocational secondary education is usually completed in four years, senior general secondary education in five years, and pre-university education in six years. We also excluded students from schools that were untraceable in the NCO dataset of the matching school year (2.5%). Where no location code was registered but we could only locate one possible match in the NCO dataset, we presumed it to be the same school. Students without personal information and data from duplicated schools were also removed (< 0.1%). Lastly, we only included schools with data on at least five students for every school year, but this was the case for all schools.

### Measurements

### Outcomes

We included two outcomes in our study: the number of days per week a student consumed *fruit*, and the number of days per week a student consumed *vegetables*. This was measured with a 7-point scale and answer categories ranged from (almost) never to every day.

### School health promotion

One of the Dutch variations of SHP is the HS program. The current study was conducted in the Netherlands and is part of a broader assessment of the program [23]. It came into existence to aid schools in fostering healthier lifestyles for better health and well-being among primary-, secondary-, and secondary vocational school students [24]. During the period of our study, secondary schools were able to acquire topic certificates for different health-related areas: nutrition, physical activity, well-being, relationships and sexuality, and smoking, alcohol, and drug prevention. A certified school fulfils several criteria regarding four pillars: health education, social and physical school environments, healthy school policy, and identifying students’ health problems [24]. Some criteria for the nutrition certificate for secondary schools are implementing a healthier canteen, having an additional water tap outside the toilets, providing education with regard to nutrition, and including nutrition in the school’s policy and informing the parents and students, and monitoring the dietary intake of the students [25]. To acquire a topic certificate, a school has to fill out a self-reported survey. Together with thematic specialists, for example from the Netherlands Nutrition Centre, the program organisation checks whether the schools fulfils all criteria. If the answers are sufficient, the school receives both the requested topic certificate, such as the nutrition certificate, and the general HS program certificate. We included several characteristics related to the HS program that were obtained from the HS organisation: *HS* (did the school have the HS program certificate in the relevant school year); *HS ever* (whether the school had been granted the HS program certificate since the start of the program (2010 [26])); *number of years HS* (the total number of school years that the school has or has had the HS program certificate as of the start of the program, up to and including the year of measurement); and the *nutrition* certificate (whether the school had the topic certificate in the relevant school year). The degree of implementation of the HS program can differ to a great extent between schools, but we utilised HS certification as an indicator of implementation adherence of the minimum requirements of the program.

### General school characteristics

We included multiple general school characteristics from the NCO dataset: *school size* (number of students); *urbanicity* of the *school area* (low (< 1000 addresses/km²), medium, and high (≥ 1500 addresses/km²); and *school type* (public, independent non-denominational, Protestant, Catholic, and collaboration/other).

### School population characteristics

We included the subsequent school population characteristics as the proportion of students in a school, based on data from the Youth Health Monitor and its precursor E-MOVO: *age* (being younger than 14, and being 14 or 15 years old. Being older than 15 was used as a reference); students in *grade* 2 (grade 4 was used as a reference); and the *educational track* (pre-university education, and senior general secondary education. Pre-vocational secondary education was used as a reference); having good *self-rated general health*; having borderline/abnormal *psychosocial health*; being *bullied at school;* being *cyberbullied*; *truancy* (skipped school at least once in the four weeks prior to the measurement); *sickness* (being absent from school due to sickness more than five days in the four weeks prior to the measurement); *school experience* (having a positive experience, and having a negative experience. Having an average experience was used as a reference.); *urbanicity* of the *home area* (low, and high, medium was used as a reference. This was based on the postal code of the home address); and *poverty level* (living in a high-poverty area). We obtained the poverty level from the NCO dataset, but the other characteristics were calculated based on the individual characteristics of the students from the health monitors. Where students specified that they were following two educational tracks (e.g. pre-vocational secondary education and senior general secondary education), which is possible in the Netherlands, we classified it as the lower track. Where all three educational tracks were specified, this was categorised as senior general secondary education. The Strengths and Difficulties Questionnaire (SDQ) [27], which is included in the more elaborate Youth Health Monitor and E-MOVO, was used to measure psychosocial health and scores could range from 0 to 40. We classified scores higher than 12 as borderline/abnormal, as was advised in the guidelines of the Netherlands Organisation for Applied Scientific Research (TNO) [28]. Self-rated general health was measured using a 5-point scale ranging from very good to (very) bad. We classified the two highest scores as good and the other three categories as less than good. A 5-point scale was also used to assess school experience, and answer categories ranged from ‘very nice’ to ‘horrible’. The two highest categories were combined to indicate a positive school experience, the middle category indicated whether their experience was average, and the two lowest categories indicated a negative school experience. Additionally, we added a school-level estimate for some school population characteristics based on an NCO dataset of students in their final year of secondary school, also expressed as the proportion of the school population: *high parental educational attainment* (having at least one higher educated parent); *household income* (high and low, medium was used as a reference.); and *migration background* (first generation and second generation, native was used as a reference.). These data were yearly available, except for the school year 2019–2020. Therefore we used the data of the school year 2018–2019 for 2019–2020. For the school year 2011–2012 we used the data of 2012–2013, since no data were available of students following the educational track pre-university education in 2011–2012. We also included the *season* the survey was filled out, i.e. fall (September until December), winter (January until March), or spring/summer (April until August), and whether the survey was filled out *anonymously* or not. Lastly, we included the *school year* as a categorical variable.

### Statistical analyses

To analyse our data, we used the 4.2.3 version of R [29]. Multiple imputation was performed to deal with missing data in outcomes and covariates using the mice package [30]. Missing values were imputed by means of (polytomous) logistic regression and predictive mean matching using five imputations and twenty iteration cycles. To improve the imputations we added the PHS, the sex of the student (male or female, only two options were provided), the other topic certificates (physical activity, well-being, smoking, alcohol and drug prevention and relationships and sexuality), and the five items of the subscale pro-social behaviour of the SDQ as auxiliary variables. The pro-social behaviour subscale is not used to calculate the total SDQ score. An auxiliary variable is a variable that is related to the variables with missing data or to the probability of missing data. The additional information from these variables can boost the imputations [31]. Another auxiliary variable was the total number of years the school received more intensive support to implement the HS program for one of the health themes, as of the school year 2015–2016. To account for differences between schools, we added the estimated variance at the school-level regarding fruit and vegetable consumption to our imputation model. We did not include variables in our imputation model that were constructed of other variables within the dataset, such as the HS indicator. The total SDQ score was imputed by using a passive imputation procedure, where the twenty item scores are imputed and the SDQ total score is re-calculated by adding up these item-level scores [32].

After the imputation process, we used the lme4 package [33] to perform multilevel regression analyses, consisting of a three-level model: students who were nested in school years, which were nested in schools. Our first step was to examine the variation between schools regarding fruit and vegetable consumption using a random intercept model (the null model). The results were used to assess the intraclass correlation coefficient (ICC), i.e. explained variation, with a possible range from 0 to 100%. The used formulas are presented in additional file 2.

In the second step of our analysis, we included school context characteristics in the null model separately to assess the change in proportion of explained variation. Variables that accounted for ≥ 10% of the variation (both at the school and the school-year level) were deemed meaningful for explaining variation between schools. These were included as confounders in the subsequent analyses [34, 35], to adjust for the influence of these characteristics [36]. In the next step, we examined the association of the HS program certificate, the number of years the school has or had been a certified school, and the nutrition certificate specifically, with the outcomes in a random intercept model. For examining of the relation between the nutrition certificate and fruit and vegetable consumption, the topic certificates were divided in three groups: (1) the nutrition certificate, (2) a different topic certificate, (3) no topic certificate. This was determined for each school year separately. The comparison between the nutrition certificate to the other topic certificates was made because students in schools with the HS program certificate in general might also be more aware of the importance of health behaviours, which may influence their dietary intake. For these analyses, we only included schools that obtained the HS program certificate within our study period, i.e. schools which were included in our dataset before and after obtaining the HS program certificate, since schools did not necessarily participate every school year. If a school’s HS program certificate was expired in 2019, this school year was not included in these analyses.

For the final step of our analysis, we used the same subset to examine whether the association between either the HS program certificate or the nutrition certificate and the outcome was different for subgroups. To examine this moderation, we added an interaction term between the HS certification and the school context characteristics that were identified as important in the ICC-analyses. A p-value of < 0.05 was used to indicate significance.

In general, all analyses were adjusted for the season of administration and whether the survey was filled out anonymously. When examining school population characteristics, adjustments were made for individual-level characteristics where possible. To assess the influence of missing data on our findings, we performed a complete case analysis and compared the results to our main findings based on imputed data. For being cyberbullied, the question was slightly different in the school years 2011–2012 and 2013–2014 compared to 2015–2016 and 2019–2020. Therefore, we also performed the ICC-analyses for being cyberbullied without the school years 2011–2012 and 2013–2014 as sensitivity analyses.

## Results

### Descriptive statistics

Table 1 offers an overview of the descriptive statistics, separately for schools that acquired the HS program certificate at least once since the start of the program (throughout the remainder of the study, we will denote these schools as ‘all certified schools’), for schools that never had the HS program certificate, and for a subsample of certified schools, i.e. schools that acquired the HS program certificate during the period of our study. In total, we included 256 secondary schools and 168,127 students in the ICC analyses. This resulted in 576 school x school year combinations. Of the 256 schools, 67 schools obtained the HS program certificate within our study period, mostly for the health themes physical activity and nutrition. We included 58,663 students in these schools. The average number of days per week students consumed fruit and vegetables was significantly different between certified and non-certified schools. The average number of days a student consumed fruit was 4.34 days in certified schools and 4.30 days in non-certified schools; the average number of days for vegetable consumption was 5.71 days in certified schools and 5.67 days in non-certified schools. There were no significant differences between all certified schools and the subsample of certified schools for our outcomes. On average, data were available of 292 students per school for every school year, representing approximately 33% of the total school population of the included schools.

**Table 1:** Descriptive statistics of secondary schools separately for (a subsample of) certified- and non-certified schools

	Certified schools¹ (*N* = 215²)	Non-certified schools¹ (*N* = 361²)	Subsample certified schools¹ (*N* = 184²)
Schools (N)	85	171	67
Students (N)	65,731	102,396	58,663
Fruit consumption (days per week)³ (Mean (SD))	4.34 (2.41)	4.30 (2.42)*	4.34 (2.40)
Vegetable consumption (days per week)³ (Mean (SD))	5.71 (1.54)	5.67 (1.57)*	5.71 (1.54)
**School health promotion**			
Number of years Healthy School (Mean (SD))	1.42 (1.86)	-*	1.40 (1.85)
*Healthy School topic certificates (yes) (%)* ^*5*^			
Nutrition	18.60	-*	20.11
Physical activity	20.93	-*	22.28
Well-being	6.98	-*	7.07
Smoking, alcohol, and drug prevention	6.98	-*	8.15
**General school characteristics**			
School size (number of students) (Mean (SD))	952 (575)	859 (506)	971 (560)
Urbanicity school area (%)			
High	48.84	40.17*	50.54
Medium	15.81	25.21*	14.13
Low	35.35	34.63	35.33
School type (%)			
Public	22.79	25.21	22.28
Independent non-denominational	11.63	12.47	12.50
Catholic	28.84	30.75	30.43
Protestant	20.93	15.79	20.65
Rest	15.81	15.79	14.13
**School population characteristics** (Mean (SD))			
Poverty level (proportion)	0.08 (0.12)	0.08 (0.12)	0.09 (0.12)
Proportion high educational attainment^7^;	0.52 (0.20)	0.51 (0.19)	0.53 (0.20)
Household income^6 7^			
Proportion low household income	0.03 (0.02)	0.03 (0.02)	0.03 (0.02)
Proportion high household income	0.57 (0.14)	0.57 (0.14)	0.58 (0.14)
Migration background^6 7^			
Proportion first generation	0.03 (0.02)	0.03 (0.03)	0.03 (0.02)
Proportion second generation	0.13 (0.08)	0.13 (0.08)	0.13 (0.08)
Age^6^			
Proportion younger than 14	0.38 (0.13)	0.36 (0.17)	0.38 (0.13)
Proportion 14–15 years	0.45 (0.09)	0.45 (0.10)	0.45 (0.08)
Grade^6^			
Proportion grade 2	0.55 (0.18)	0.52 (0.23)	0.55 (0.17)
Educational track^6^			
Proportion pre-university education	0.20 (0.26)	0.19 (0.26)	0.21 (0.26)
Proportion senior general secondary education	0.24 (0.24)	0.23 (0.25)	0.25 (0.24)
Proportion good self-rated general health	0.86 (0.05)	0.85 (0.05)	0.86 (0.04)
Proportion borderline/abnormal psychosocial health	0.26 (0.07)	0.27 (0.08)	0.26 (0.07)
Proportion bullied at school (yes)^10^	0.11 (0.05)	0.11 (0.05)	0.11 (0.04)
Proportion cyberbullied (yes)^9^	0.06 (0.03)	0.06 (0.03)	0.06 (0.03)
Proportion truancy (yes)^8^	0.09 (0.05)	0.10 (0.06)*	0.09 (0.04)
Proportion sickness (more than five days)_8_	0.04 (0.03)	0.04 (0.03)	0.04 (0.03)
School experience^6 11^			
Proportion positive	0.54 (0.09)	0.52 (0.10)	0.54 (0.09)
Proportion negative	0.10 (0.04)	0.10 (0.04)	0.09 (0.04)
Urbanicity home area^6 12^			
Proportion high urbanicity	0.35 (0.28)	0.32 (0.30)	0.35 (0.28)
Proportion low urbanicity	0.49 (0.31)	0.49 (0.33)	0.49 (0.31)

*Note* - = Not applicable. * differs significantly from certified schools (*p* < 0.05). ^1^ Certified schools acquired the HS program certificate at least once, non-certified schools never, and the certified schools subsample has data before and after obtaining the HS program certificate. ^2^ N (school x school year combinations) with information unless specified otherwise. We summarised results per school, for each school year individually. ^3^ Outcomes are on the individual-level. For fruit consumption, data were missing of 3953 students in all certified school, 4862 students in non-certified schools, and 3648 students in the certified schools subsample. For vegetable consumption, data were missing of 3984 students in all certified school, 4865 students in non-certified schools, and 3679 students in the certified schools subsample. ^4^ N (school x school year combinations): 358 for non-certified schools. ^5^ Due to privacy, percentages are not presented for the topic certificate relationships and sexuality. ^6^ The following were used as a reference (as proportion of students): medium household income, native, older than 15, grade 4, pre-vocational secondary education, average school experience, and living in a medium urbanicity. ^7^ N (school x school year combinations): 208 for certified schools, 330 for non-certified schools and 178 for the certified schools subsample. ^8^ N (school x school year combinations): 192 for certified schools, 325 for non-certified schools, and 164 for the certified schools subsample. ^9^ N (school x school year combinations): 177 for certified schools, 301 for non-certified schools and 155 for the certified schools subsample. ¹_10_ N (school x school year combinations): 356 for non-certified schools. ¹^11^ N (school x school year combinations): 197 for certified schools, 337 for non-certified schools and 169 for the certified schools subsample. ^12^ N (school x school year combinations): 360 for non-certified schools. SD = Standard deviation

### Differences in fruit consumption

Table 2 shows the results of the ICC-analyses for fruit consumption including all schools.

Our results indicate that differences between schools accounted for 2.87% of the total variation in fruit consumption. Nine characteristics explained ≥ 10% of this variation: high parental educational attainment, household income, the educational track of the students, age, being bullied at school, being cyberbullied, self-rated general health, psychosocial health, and school experience. High parental educational attainment, the educational track of the students, and the household income explained most of the variance. Students in schools with relatively more high educated parents, more students following senior general secondary education or pre-university education, or more students with a high household income consumed fruit on more days. Fruit consumption within schools differed for less than 1% over school years, i.e. 0.78%. Seven characteristics explained this variation over time within schools, i.e. the HS program certificate, the number of years the school has or had been a certified school, high parental educational attainment, age, being bullied at school, being cyberbullied, and sickness.

Table 3 presents the results for the analyses examining the association between HS certification and fruit consumption, adjusted for all characteristics that accounted for ≥ 10% of the variation between schools and school years, as well as the season and whether the survey was filled out anonymously. For these analyses, we only included schools that obtained the HS program certificate within our study period. When schools had the HS program certificate, students consumed fruit on more days, compared to when schools did not have the HS program certificate (B = 0.15, 95% CI: 0.05, 0.24). More specifically, students in schools with the nutrition certificate also consumed fruit on more days, in comparison to students in schools without the HS program certificate (B = 0.20, 95% CI: 0.10, 0.31). However, there was no significant difference between students in schools with the nutrition certificate and students in schools with other topic certificates.

Additionally, we also found a significant positive association between the number of years a school has or had the HS program certificate, and the number of days a student consumed fruit (B = 0.06, 95% CI: 0.03, 0.09). However, some school population characteristics moderated the association between the HS program certificate and fruit consumption of students [see Table S1 in Additional file 3]: e.g. the proportion of students with an abnormal or borderline SDQ-score: an increase of 10% led to an estimated favourable difference of 0.12 days. The same accounted for the proportion of students that had been absent from school due to sickness for more than five days prior to the measurement, an increase of 10% led to an estimated favourable difference in the association of 0.37 days. For school experience, we also found a different association for schools with a higher share of students with a negative experience instead of having an average experience. An increase of 10% in students with a negative school experience led to an estimated favourable difference of 0.44 days.

### Differences in vegetable consumption

As presented in Table 2, differences between schools were accounted for 5.57% of the total variation in vegetable consumption, when including all schools. Eight characteristics explained ≥ 10% of this variation, i.e. high parental educational attainment, household income, migration background, the educational track of the students, self-rated general health, being bullied at school, being cyberbullied, and school experience. High parental educational attainment, the educational track of the students, and the household income explained most of the variance. Students in schools with relatively more high educated parents, more students following senior general secondary education or pre-university education, or more students with a high household income consumed vegetables on more days. Vegetable consumption within schools differed for less than 1% over school years, i.e. 0.77%. Eight characteristics explained this variation over time within schools, i.e. the HS program certificate, the number of years a school has or had been a certified school, high parental educational attainment, being bullied at school, the educational track of the students, self-rated general health, being cyberbullied, and sickness. Table 3 presents the results for the analyses examining the association between HS certification and vegetable consumption. When considering the schools that obtained the HS program within our study period, students consumed vegetables on more days, compared to when schools did not have the HS program certificate (B = 0.07, 95% CI: 0.02, 0.13). We also found a positive association between the number of years a school has or had been a certified school and the number of days a student consumed vegetables (B = 0.03, 95% CI: 0.02, 0.05). When examining the differences between the topic certificates, students in schools with the nutrition certificate consumed vegetables slightly more often compared to students in schools with other topic certificates (B = 0.09, 95% CI: 0.02, 0.16) or no HS program certificate (B = 0.12, 95% CI: 0.06, 0.18), indicating that there is a favourable association between the nutrition certificate and vegetable consumption of students.

For vegetable consumption, only migration background moderated the association between the nutrition certificate and the number of days a student consumed vegetables [see Table S2 in Additional file 3]. An increase of 10% in the proportion of students with a second migration background instead of having a native background, led to an estimated differences of 0.09 days.

**Table 2:** Multilevel intraclass correlations in secondary schools for fruit and vegetable consumption

	Fruit¹ (*N* = 576²)		Vegetables¹ (*N* = 576²)	
	ICCschool-level (%)	ICC school year-level (%)	ICC school-level (%)	ICC school year-level (%)
0 model	2.87	0.78	5.57	0.77
**School health promotion**				
Healthy School	2.90	0.69*	5.61	0.67*
Healthy School ever	2.88	0.78	5.58	0.77
Number of years Healthy School	2.94	0.61*	5.63	0.63*
Nutrition	2.94	0.71	5.60	0.70
**General school characteristics**				
School size	2.58	0.83	5.11	0.83
Urbanicity school area	2.85	0.75	5.65	0.72
School type	2.83	0.78	5.56	0.77
**School population characteristics**				
Poverty level	2.82	0.77	5.10	0.77
High parental educational attainment	0.63*	0.52*	1.84*	0.45*
Household income³	1.00*	0.79	2.96*	0.84
Migration background³	2.72	0.78	4.93*	0.79
Age_3 4_	2.27*	0.64*	5.11	0.73
Grade_4_	2.85	0.73	5.57	0.77
Educational track^3 4^;	0.99*	0.73	2.53*	0.66*
Self-rated general health^4^	2.46*	0.71	5.01*	0.63*
Psychosocial health^4^	2.54*	0.87	5.21	0.83
Bullied at school^4^	2.25*	0.57*	5.00*	0.46*
Cyberbullied^4^	2.38*	0.66*	4.95*	0.46*
Truancy^4^	2.88	0.79	5.60	0.78
Sickness^4 5^	3.22	0.67*	6.09	0.66*
School experience^3 4^	1.86*	0.80	4.34*	0.72
Urbanicity home area^3 4^	2.92	0.75	5.65	0.76

*Note* All analyses were controlled for the season of administration and whether the survey was filled out anonymously or not. ^1^ Included as number of days per week consumed. ^2^ Number of school x school year combinations. N (schools) = 256, N (students) = 168,127. * Adding the characteristic to the 0-model reduced the ICC by ≥ 10%. ^3^ The school population characteristic consists of two variables. ^4^ The analysis was controlled for the individual characteristic. ^5^ Due to convergence warnings, we changed the optimizer method for the model estimation

**Table 3:** Association between Healthy School certification and dietary intake of students in secondary schools

	Fruit¹ (*N* = 184²)		Vegetables¹ (*N* = 184²)	
	B	95% CI	B	95% CI
**Model 1**				
Intercept	2.40	(1.14, 3.66)*	5.09	(4.43, 5.75)*
HS	0.15	(0.05, 0.24)*	0.07	(0.02, 0.13)*
**Model 2**				
Intercept	2.55	(1.32, 3.77)*	5.08	(4.45, 5.71)*
Number of years HS	0.06	(0.03, 0.09)*	0.03	(0.02, 0.05)*
**Model 3**				
Intercept³	2.60	(1.33, 3.87)*	5.17	(4.51, 5.82)*
No HS	-0.20	(-0.31, -0.10)*	-0.12	(-0.18, -0.06)*
HS, but no nutrition certificate	-0.10	(-0.21, 0.01)	-0.09	(-0.16, -0.02)*

*Note* ¹ Expressed as number of days per week consumed. ² N = Number of schools x school year combinations. ³ Having the nutrition certificate is used as a reference group. * = Significant (*p* < 0.05. N (schools) = 67; N (students) = 58,663. All analyses were controlled for the season of administration and whether the survey was filled out anonymously or not. We adjusted for all characteristics that accounted for ≥ 10% of the variation between schools in Table 2, as well as the individual characteristic, which were not related to the HS program. Regression coefficients for these control variables are not displayed in the table. For the control variables, we used the following as a reference for the school population and individual characteristics: being older than 15 (only for fruit), following pre-vocational secondary education, having less than good self-rated general health, having normal psychosocial health (only for fruit), not being bullied at school, not being cyberbullied, not being absent from school due to sickness more than five days, having an average school experience, and filling out the survey anonymously during fall. HS = the HS program certificate; CI = confidence interval

## Discussion

This study focused on examining differences between secondary schools in the Netherlands regarding fruit and vegetable consumption of students, and to what extent these differences can be explained by differences between schools regarding HS certification, general school characteristics, and school population characteristics. Fruit and vegetable consumption were examined separately. We also examined the relation between HS certification and fruit and vegetable consumption, and whether this association was moderated by contextual school factors. Our results suggested that variation in fruit and vegetable consumption by students is mostly explained by individual characteristics, but that a small part of these dietary behaviours are due to characteristics at the school-level.

For both outcomes, characteristics related to high parental educational attainment, the educational track of the students, and household income were most important in explaining differences in fruit and vegetable consumption between schools. This is in line with literature findings that showed that (indicators of) socioeconomic status, both on the individual and school-level, are associated with dietary intake [37–40]. One explanation might be that families with lower household incomes might have difficulties to afford buying fruit and vegetables [41–43]. Another study showed that children’s knowledge of the fruit and vegetable recommendations was an important mediating factor between parental educational attainment and fruit and vegetable consumption of children [44]. The educational track of students was also identified as an important characteristic in the national results of the Youth Health Monitor of 2015 [45]. Results of previous research in 21 European countries also indicated that a higher educational level of people of 15 and over was positively related to fruit and vegetable consumption [46].

For fruit and vegetable consumption, variation within schools was even smaller than variation between schools. Even though variation within schools was small, we found that it is partly explained by schools obtaining the HS program certificate and the duration of being a certified school. When schools had the HS program certificate, students consumed fruit on slightly more days, compared to when schools did not have the HS program certificate. The same accounted for vegetables. For both outcomes, the association with the HS program certificate appeared to become stronger over time. Our results showed that fulfilling the requirements of the HS program, especially with a focus on nutrition, might have a small positive effect on fruit and vegetable consumption by students. This is in line with the results of a systematic review that showed that interventions solely focusing on nutrition had a small, favourable effect on fruit and vegetable consumption of students [14].

Moreover, the HS program may actually be especially advantageous in specific school populations. For fruit consumption, we found stronger favourable associations when schools had relatively more students with an abnormal or borderline SDQ-score, relatively more students who had a negative school experience, or relatively more students who had been absent from school due to sickness for at least five days in the four weeks prior to the measurement. To our knowledge, limited literature is currently available about the interaction between these school population characteristics and SHP. Nevertheless, exposure to a health-promoting intervention or program is important for behaviour change [47]. Therefore, it could be expected that students who are more often absent due to sickness have less exposure time to the HS program, and therefore receive a lower dose, which might weaken the association. However, our results did not support this. Another study on school satisfaction showed that having lower school satisfaction could inhibit the impact of health promotion, since these students were less likely to follow the advice from school health nurses or to discuss it with their parents [48]. Worse psychosocial health or mental health has also been consistently associated with worse dietary intake in students [49]. Based on the current study, no explanations can be provided for the found moderations. It is possible that these findings are due to unobserved confounders, but future research should further examine these moderations. For vegetable consumption, we found a stronger association for the nutrition certificate for schools with relatively more students with a second generation migration background compared to a native background. A possible explanation is that students in schools with relatively more students with a second generation migration background consume on average vegetables less often than native students, as shown by our results. Therefore, leaving more room for improvement. This was in line with results from another study in the Netherlands, that showed that children with a non-western migration background were more likely to have low vegetable consumption [50].

### Implications for practice and research

Obtaining the HS program certificate, and especially the nutrition certificate, seems to contribute to a slightly higher consumption of fruit and vegetables of secondary school students. Therefore, we recommend to schools aiming to promote fruit and vegetable intake of students, to obtain the nutrition certificate of the HS program. The HS organisation should keep prioritising schools with a lower SES, as well as schools with a relatively high share of students following the educational track pre-vocational secondary education, since students in these schools are more likely to consume fruit and vegetable less frequent. Additionally, we found small differences for some subgroups, but future research should focus on the impact in different school contexts, since we were restricted in the characteristics that could be included in this study. Other potentially important contextual factors are for example parental involvement [51] and the degree of implementation of the HS program [52]. The degree of implementation of the HS program can vary to a great extent between schools, and future studies should explore its influence [52].

### Strengths and limitations

Since we used existing datasets, we were able to include a large number of schools in our analyses. Therefore, we could perform multilevel analyses and contribute to the currently existing literature with regard to the relation between SHP and fruit and vegetable consumption. Since we included a large number of schools and students in our analyses, we assume the generalisability and external validity of our results to be high. Additionally, the performed complete-case analysis and sensitivity analysis confirmed the robustness of our results.

Nevertheless, the current study also had a few limitations. Firstly, we were not able to include the degree of implementation of the HS program, but schools without the HS program certificate might also have implemented the HS program or other SHP programs. Secondly, the question for being cyberbullied was slightly different in different school years, this might have resulted in some information bias. Additionally, since not every PHS included the same questions in their survey, we had to impute some variables for the entire school in some school years. However, we performed sensitivity- and complete case analyses to examine the possible impact of the information bias and missing values and these led to similar conclusions. Lastly, our outcomes, as well as some school population characteristics, were self-reported. Since students could give desirable answers, this might have also caused some information bias [53]. It is possible that students in schools with the HS program certificate or the nutrition certificate are more prone to this, which might have overestimated the associations. However, most surveys were filled out anonymously which decreased this risk. We also adjusted in our analyses whether the survey was filled out anonymously or not. Lastly, the HS system is updated if schools merge or split. It is therefore plausible that we were not able to identify all schools with the HS program certificate in our dataset, which might have caused non-differential bias [54].

## Conclusions

The variation between schools regarding fruit and vegetable consumption of students was mostly explained by high parental educational attainment, household income, and the educational track of students. Even though schools did not seem to play a large role in fruit and vegetable consumption of students, we found that obtaining HS certification, and especially the nutrition certificate for vegetable consumption, might contribute to slightly healthier lifestyles regarding these behaviours. We observed small differences for some subgroups, but future research should focus on the impact in different school contexts, since we were restricted in the contextual factors that could be included in this study, and on the effect of the HS program in primary schools and secondary vocational schools.

*Results based on calculations by Maastricht University using non-public microdata from Statistics Netherlands. Under certain conditions, these microdata are accessible for statistical and scientific research. For further information: microdata@cbs.nl*.

### Electronic supplementary material

Below is the link to the electronic supplementary material.

Supplementary Material 1

Supplementary Material 2

Supplementary Material 3

## Data Availability

The majority of the data that supports the findings of this study are available from the Association of Regional Public Health Services (GGD GHOR), Statistics Netherlands and Healthy School. However, restrictions apply to the availability of these data, which were used under licence for the current study, and are not publicly available. (Micro)data from Statistics Netherlands, the Health Monitor Youth 2019 and Healthy School are however accessible for statistical and scientific research under criteria defined by the organisations [55-57]. To request the data from this study, you can contact monitorgezondheid@ggdghor.nl (GGD GHOR), microdata@cbs.nl (Statistics Netherlands) and/or info@gezondeschool.nl (Healthy School).

## Abbreviations

CI: confidence interval
E-MOVO: Electronic Monitor and Health Education
GGD GHOR: the Association of Regional Public Health Services
Havo: senior general secondary education
HPS: Health Promoting Schools
HS: Healthy School
ICC: intraclass correlation coefficient
N/no: number
NCO: the Netherlands Cohort Study on Education
NRO: the Netherlands Initiative for Education Research
PHS: Public Health Service
SD: standard deviation
SDQ: the Strengths and Difficulties Questionnaire
SHP: school health promotion
TNO: the Netherlands Organisation for Applied Scientific Research
Vmbo: pre-vocational secondary education
Vwo: pre-university education
WHO: World Health Organization
