# Supplementary material for: School health promotion and fruit and vegetable consumption in secondary schools: a repeated cross-sectional multilevel study
Source: BMC Public Health. 2024 Apr 22;24:1098. doi: 10.1186/s12889-024-18546-2 (PMC11034157; doi:10.1186/s12889-024-18546-2)
Supplement: Supplementary file 1 — Supplementary Material 1 [file 12889_2024_18546_MOESM1_ESM.docx]

**Additional file 1**

File name: Additional file 1
File format: .pdf
Title of data: Overview of included variables
Description of data: Overview of included variables in the survey separately per Public Health Service and school year.

**Table 1: Overview of included variables in the survey separately per Public Health Service and school year**

| *Variable* | **GGD Gelderland-Midden** | | | **GGD Gelderland-Zuid** | | | **GGD IJsselland** | | | **GGD Kenne-merland** | | | **GGD Limburg-Noord** | | | **GGD Noord- en Oost-Gelderland** | | | **GGD Zuid Limburg** | | |
| --- | --- | --- | --- | --- | --- | --- | --- | --- | --- | --- | --- | --- | --- | --- | --- | --- | --- | --- | --- | --- | --- |
| *Year of survey* | 11 | 15 | 19 | 11 | 15 | 19 | 13 | 15 | 19 | 13 | 15 | 19 | 13 | 15 | 19 | 11 | 15 | 19 | 13 | 15 | 19 |
| Fruit | x | x | - | x | x | x | x | x | - | x | x | x | x | x | x | x | x | x | x | x | x |
| Vegetables | x | x | - | x | x | x | x | x | - | x | x | x | x | x | x | x | x | x | x | x | x |
| Age | x | x | x | x | x | x | x | x | x | x | x | x | x | x | x | x | x | x | x | x | x |
| Grade | x | x | x | x | x | x | x | x | x | x | x | x | x | x | x | x | x | x | x | x | x |
| Educational track | x | x | x | x | x | x | x | x | x | x | x | x | x | x | x | x | x | x | x | x | x |
| Urbanicity home area (based on postal code) | x | x | x | x | x | x | x | x | x | x | x | x | x | x | x | x | x | x | x | x | x |
| School experience | x | - | - | x | x | x | x | x | - | x | x | x | x | x | x | x | x | x | x | x | x |
| Sickness | x | x | - | x | x | x | x | x | - | x | x | - | x | x | x | x | x | x | x | x | x |
| Cyberbullied | - | x | x | # | x | x | - | x | x | x | x | x | # | x | x | # | x | x | # | x | x |
| Bullied at school | x | x | x | x | x | x | x | x | x | x | x | x | x | x | x | x | x | x | x | x | x |
| Self-rated general health | x | x | x | x | x | x | x | x | x | x | x | x | x | x | x | x | x | x | x | x | x |
| Psychosocial health | x | x | x | x | x | x | x | x | x | x | x | x | x | x | x | x | x | x | x | x | x |
| Truancy | x | x | - | x | x | x | x | x | - | x | x | - | x | x | x | x | x | x | x | x | x |

Note: 11 = survey of 2011-2012, 13 = survey of 2013-2014, 15 = survey of 2015-2016, and 19 = survey of 2019-2020; X = variable was included in the survey; - = variable was not included in the survey; # = slightly different question was included in the survey (time period differed)
