# Supplementary material for: School health promotion and fruit and vegetable consumption in secondary schools: a repeated cross-sectional multilevel study
Source: BMC Public Health. 2024 Apr 22;24:1098. doi: 10.1186/s12889-024-18546-2 (PMC11034157; doi:10.1186/s12889-024-18546-2)
Supplement: Supplementary file 2 — Supplementary Material 2 [file 12889_2024_18546_MOESM2_ESM.docx]

**Additional file 2**File name: Additional file 2
File format: .pdf
Title of data: Formulas ICC-analyses
Description of data: Formulas ICC-analyses

${ICC}_{school}= \frac{\sigma_{school}^{2}}{\sigma_{school}^{2}+ \sigma_{school year}^{2}+\varepsilon}$ x 100%

${ICC}_{schoolyear}= \frac{\sigma_{school year}^{2}}{\sigma_{school}^{2}+ \sigma_{school year}^{2}+\varepsilon}$ x 100%

The formulas display the estimated variance at the school-level and the school year-level as $\sigma_{school}^{2}$ and $\sigma_{school year}^{2}$. The residual variance is displayed as $\varepsilon$.
