# Supplementary material for: School health promotion and fruit and vegetable consumption in secondary schools: a repeated cross-sectional multilevel study
Source: BMC Public Health. 2024 Apr 22;24:1098. doi: 10.1186/s12889-024-18546-2 (PMC11034157; doi:10.1186/s12889-024-18546-2)
Supplement: Supplementary file 3 — Supplementary Material 3 [file 12889_2024_18546_MOESM3_ESM.docx]

**Additional file 3**

File name: Additional file 3
File format: .pdf
Title of data: Interaction analyses
Description of data: Interaction between HS certification and school population characteristics on fruit and vegetable consumption.

*Table S1: Interaction between the HS program certificate and school population characteristics on fruit consumption*

|  |  |  |
| --- | --- | --- |
|  | **Fruit¹ (N = 184²)** | |
|  | **B** | **95% CI** |
| *Model 1:* |  |  |
| Intercept | 2.34 | (1.08, 3.60)* |
| High educational attainment | 1.01 | (0.36, 1.66)* |
| HS | 0.27 | (0.06, 0.48)* |
| HS x high educational attainment | -0.21 | (-0.54, 0.12) |
| *Model 2:* |  |  |
| Intercept | 2.26 | (0.99, 3.53)* |
| Low household income | 1.01 | (-1.77, 3.78) |
| High household income | 0.87 | (0.15, 1.59)* |
| HS | 0.25 | (-0.17, 0.68) |
| HS x low household income | 2.19 | (-2.35, 6.73) |
| HS x high household income | -0.28 | (-0.83, 0.28) |
| *Model 3:* |  |  |
| Intercept | 2.11 | (0.71, 3.51)* |
| Younger than 14 | 0.21 | (-0.36, 0.78) |
| 14-15 years old | 0.26 | (-0.64, 1.16) |
| HS | 0.96 | (-0.20, 2.12) |
| HS x Younger than 14 | -1.21 | (-2.95, 0.53) |
| HS x 14-15 years old | -0.74 | (-1.91, 0.44) |
| *Model 4:* |  |  |
| Intercept | 2.38 | (1.12, 3.64)* |
| Havo | -0.12 | (-0.44, 0.21) |
| Vwo | -0.03 | (-0.40, 0.33) |
| HS | 0.20 | (0.06, 0.33)* |
| HS x havo | -0.04 | (-0.32, 0.23) |
| HS x vwo | -0.15 | (-0.44, 0.14) |
| *Model 5:* |  |  |
| Intercept | 2.06 | (0.75, 3.36)* |
| Good self-rated general health | 0.22 | (-0.90, 1.34) |
| HS | 1.32 | (-0.33, 2.97) |
| HS x good self-rated general health | -1.34 | (-3.22, 0.54) |
| *Model 6:* |  |  |
| Intercept | 2.54 | (1.30, 3.77)* |
| Abnormal/borderline psychosocial health | -0.03 | (-1.12, 1.05) |
| HS | -0.15 | (-0.43, 0.14) |
| HS x abnormal/borderline psychosocial health | 1.19 | (0.12, 2.25)* |
| *Model 7:* |  |  |
| Intercept | 2.42 | (1.16, 3.68)* |
| Being bullied at school | -1.37 | (-3.18, 0.44) |
| HS | 0.08 | (-0.13, 0.28) |
| HS x being bullied at school | 0.70 | (-1.13, 2.54) |
| *Model 8:* |  |  |
| Intercept | 2.48 | (1.23, 3.74)* |
| Being cyberbullied | 0.34 | (-1.50, 2.19) |
| HS | 0.04 | (-0.14, 0.21) |
| HS x being cyberbullied | 2.20 | (-0.82, 5.21) |
| *Model 9:* |  |  |
| Intercept | 2.43 | (1.18, 3.68)* |
| Sickness | -3.21 | (-6.56, 0.13) |
| HS | 0.04 | (-0.10, 0.17) |
| HS x sickness | 3.67 | (0.31, 7.02)* |
| *Model 10:* |  |  |
| Intercept | 2.38 | (1.14, 3.61)* |
| Positive school experience | 0.08 | (-0.72, 0.87) |
| Negative school experience | -0.89 | (-2.66, 0.88) |
| HS | -0.49 | (-1.49, 0.51) |
| HS x positive school experience | 0.40 | (-0.96, 1.76) |
| HS x negative school experience | 4.37 | (1.02, 7.72)* |

*Note: Results are based on schools that are included in our dataset before and after obtaining the HS program certificate. All analyses have been adjusted for the season of administration, whether the survey was filled out anonymously or not, and characteristics that accounted for > 10% of the variation between schools in Table 2 as well as the individual characteristic, which were not related to the HS program. Results for control variables are not displayed in the table. For the control variables, we used the following as a reference for the school population and individual characteristics: being older than 15, following vmbo, having less than good self-rated general health, having normal psychosocial health, not being bullied at school, not being cyberbullied, not being absent from school due to sickness more than five days, having an average school experience, and filling out the survey anonymously during fall. All school population characteristics are included in the analyses as proportion. ¹ Expressed as number of days per week consumed. ² N = Number of schools x school year combinations. In total, 67 schools and 58663 pupils are included in the analyses. * = p-value < 0.05. CI = confidence interval; HS = Healthy School program certificate*

*Table S2: Interaction between HS certification and school population characteristics on vegetable consumption*

|  | **Vegetable¹ (N = 184²)** | |
| --- | --- | --- |
|  | **B** | **95% CI** |
| *Model 1:* |  |  |
| Intercept⁴ | 5.03 | (4.38, 5.68)* |
| High educational attainment | 0.87 | (0.41, 1.33)* |
| Nutrition certificate | 0.08 | (-0.09, 0.26) |
| HS but no nutrition certificate | -0.03 | (-0.19, 0.13) |
| Nutrition certificate x high educational attainment | 0.07 | (-0.21, 0.34) |
| HS but no nutrition certificate x high educational attainment | 0.10 | (-0.15, 0.36) |
| *Model 2:* |  |  |
| Intercept⁴ | 5.01 | (4.36, 5.66)* |
| Low household income | 0.88 | (-0.85, 2.60) |
| High household income | -0.25 | (-0.74, 0.24) |
| Nutrition certificate | 0.29 | (-0.05, 0.63) |
| HS but no nutrition certificate | -0.09 | (-0.37, 0.20) |
| Nutrition certificate x low household income | 1.15 | (-1.78, 4.07) |
| Nutrition certificate x high household income | -0.20 | (-0.65, 0.25) |
| HS but no nutrition certificate x low household income | -2.01 | (-5.42, 1.41) |
| HS but no nutrition certificate x high household income | 0.17 | (-0.23, 0.56) |
| *Model 3:* |  |  |
| Intercept⁴ | 5.07 | (4.43, 5.71)* |
| First generation migration background | 0.02 | (-2.31, 2.35) |
| Second generation migration background | -0.91 | (-1.54, -0.29)* |
| Nutrition certificate | 0.06 | (-0.06, 0.18) |
| HS but no nutrition certificate | -0.03 | (-0.15, 0.09) |
| Nutrition certificate x first generation migration background | -2.50 | (-5.17, 0.18) |
| Nutrition certificate x second generation migration background | 0.90 | (0.02, 1.77)* |
| HS but no nutrition certificate x first generation migration background | 1.90 | (-1.71, 5.50) |
| HS but no nutrition certificate x second generation migration background | 0.11 | (-0.79, 1.01) |
| *Model 4:* |  |  |
| Intercept⁴ | 5.01 | (4.33, 5.68)* |
| Havo | -0.02 | (-0.26, 0.21) |
| Vwo | -0.11 | (-0.35, 0.13) |
| Nutrition certificate | 0.12 | (0.02, 0.22)* |
| HS but no nutrition certificate | -0.01 | (-0.11, 0.08) |
| Nutrition certificate x havo | -0.05 | (-0.34, 0.25) |
| Nutrition certificate x vwo | 0.06 | (-0.32, 0.43) |
| HS but no nutrition certificate x havo | 0.03 | (-0.16, 0.23) |
| HS but no nutrition certificate x vwo | 0.13 | (-0.07, 0.33) |
| *Model 5:* |  |  |
| Intercept⁴ | 4.96 | (4.27, 5.64)* |
| Good self-rated general health | 0.15 | (-0.46, 0.76) |
| Nutrition certificate | 0.35 | (-0.92, 1.62) |
| HS but no nutrition certificate | 0.60 | (-0.85, 2.05) |
| Nutrition certificate x good self-rated general health | -0.25 | (-1.73, 1.22) |
| HS but no nutrition certificate x good self-rated general health | -0.65 | (-2.29, 1.00) |
| *Model 6:* |  |  |
| Intercept⁴ | 4.98 | (4.34, 5.61)* |
| Being bullied at school | 0.48 | (-0.63, 1.59) |
| Nutrition certificate | 0.14 | (0.01, 0.28)* |
| HS but no nutrition certificate | 0.13 | (-0.05, 0.31) |
| Nutrition certificate x being bullied at school | -0.16 | (-1.49, 1.17) |
| HS but no nutrition certificate x being bullied at school | -1.02 | (-2.76, 0.73) |
| *Model 7:* |  |  |
| Intercept⁴ | 5.03 | (4.39, 5.67)* |
| Being cyberbullied | -1.30 | (-2.34, -0.27)* |
| Nutrition certificate | 0.15 | (0.00, 0.29)* |
| HS but no nutrition certificate | 0.05 | (-0.10, 0.19) |
| Nutrition certificate x being cyberbullied | -0.48 | (-3.09, 2.13) |
| HS but no nutrition certificate x being cyberbullied | -0.28 | (-2.93, 2.36) |
| *Model 8:* |  |  |
| Intercept⁴ | 5.04 | (4.38, 5.69)* |
| Sickness | -0.65 | (-2.70, 1.40) |
| Nutrition certificate | 0.11 | (0.02, 0.20)* |
| HS but no nutrition certificate | -0.01 | (-0.12, 0.09) |
| Nutrition certificate x sickness | 0.72 | (-1.26, 2.71) |
| HS but no nutrition certificate x sickness | 1.46 | (-1.24, 4.15) |
| *Model 9³:* |  |  |
| Intercept⁴ | 5.17 | (4.52, 5.81)* |
| Positive school experience | -0.38 | (-0.88, 0.11) |
| Negative school experience | -0.70 | (-1.79, 0.39) |
| Nutrition certificate | -0.45 | (-1.20, 0.30) |
| HS but no nutrition certificate | -0.36 | (-1.19, 0.47) |
| Nutrition certificate x positive school experience | 0.68 | (-0.34, 1.71) |
| Nutrition certificate x negative school experience | 2.17 | (-0.47, 4.80) |
| HS but no nutrition certificate x positive school experience | 0.40 | (-0.79, 1.60) |
| HS but no nutrition certificate x negative school experience | 1.94 | (-0.65, 4.53) |

*Note: Results are based on schools that are included in our dataset before and after obtaining the HS program certificate. All analyses have been adjusted for the season of administration, whether the survey was filled out anonymously or not, and characteristics that accounted for > 10% of the variation between schools in Table 2, as well as the individual characteristic, which were not related to the HS program. Regression coefficients for control variables are not displayed in the table. For the control variables, we used the following as a reference for the school population and individual characteristics: following vmbo, having less than good self-rated general health, not being bullied at school, not being cyberbullied, not being absent from school due to sickness more than five days, having an average school experience, and filling out the survey anonymously during fall. All school population characteristics are included in the analyses as proportion.* *¹ Expressed as number of days per week consumed. ² N = Number of schools x school year combinations. In total, 67 schools and 58663 pupils are included in the analyses. ³ Due to convergence warnings, we used a different optimizer method for the model estimation. ⁴ Having no HS program certificate is used as a reference group. * = p-value < 0.05. CI = confidence interval; HS = Healthy School program certificate*
